# Short term non-invasive ventilation post-surgery improves arterial blood-gases in obese subjects compared to supplemental oxygen delivery - a randomized controlled trial

**DOI:** 10.1186/1471-2253-11-10

**Published:** 2011-05-23

**Authors:** Martin Zoremba, Gerald Kalmus, Domenique Begemann, Leopold Eberhart, Norbert Zoremba, Hinnerk Wulf, Frank Dette

**Affiliations:** 1 Department of Anaesthesia and Intensive Care Medicine, University of Marburg, D-35033 Marburg, Germany; 2 Department of Anaesthesia and Intensive Care Medicine, University of Aachen D-52074 Aachen, Germany

**Keywords:** Obesity, atelectasis, lung function, NIV, PACU

## Abstract

**Background:**

In the immediate postoperative period, obese patients are more likely to exhibit hypoxaemia due to atelectasis and impaired respiratory mechanics, changes which can be attenuated by non-invasive ventilation (NIV). The aim of the study was to evaluate the duration of any effects of early initiation of short term pressure support NIV vs. traditional oxygen delivery via venturi mask in obese patients during their stay in the PACU.

**Methods:**

After ethics committee approval and informed consent, we prospectively studied 60 obese patients (BMI 30-45) undergoing minor peripheral surgery. Half were randomly assigned to receive short term NIV during their PACU stay, while the others received routine treatment (supplemental oxygen via venturi mask). Premedication, general anaesthesia and respiratory settings were standardized. We measured arterial oxygen saturation by pulse oximetry and blood gas analysis on air breathing. Inspiratory and expiratory lung function was measured preoperatively (baseline) and at 10 min, 1 h, 2 h, 6 h and 24 h after extubation, with the patient supine, in a 30 degrees head-up position. The two groups were compared using repeated-measure analysis of variance (ANOVA) and t-test analysis. Statistical significance was considered to be P < 0.05.

**Results:**

There were no differences at the first assessment. During the PACU stay, pulmonary function in the NIV group was significantly better than in the controls (p < 0.0001). Blood gases and the alveolar to arterial oxygen partial pressure difference were also better (p < 0.03), but with the addition that overall improvements are of questionable clinical relevance. These effects persisted for at least 24 hours after surgery (p < 0.05).

**Conclusion:**

Early initiation of short term NIV during in the PACU promotes more rapid recovery of postoperative lung function and oxygenation in the obese. The effect lasted 24 hours after discontinuation of NIV. Patient selection is necessary in order to establish clinically relevant improvements.

**Trial Registration#:**

DRKS00000751; http://www.germanctr.de

## Background

Acute respiratory failure is a major complication within the early post-operative period [1]. In patients with hypercapnic [2] or non-hypercapnic [3] acute respiratory failure, non-invasive ventilation (NIV) can reduce intubation rate, morbidity, mortality and the overall and intensive care unit (ICU) lengths of stay. NIV is thus well established in clinical practice. It is mostly applied in an intensive care setting; only a few studies have evaluated its use within the first 24 postoperative hours [4,5], although the optimum duration of treatment and ventilator settings are not defined [6,7]. Nevertheless it seems feasible that early initiation of a NIV therapy immediately after surgery may be beneficial, when residual drug effects are still present. Obesity presents a particular problem; increased BMI correlates with loss of perioperative functional residual capacity (FRC), expiratory reserve volume (ERV) and total lung capacity (TLC), up to 50% of preoperative values [8]. These changes promote atelectasis and an increased Va/Q mismatch [9] resulting in increased work of breathing. Additionally obese patients are more likely to exhibit upper airway collapse [10]. Most of the events leading up to respiratory complications occur in the post-anaesthesia care unit (PACU) [11].

Obese patients are often scheduled for day case surgery, thus it is important to promote pulmonary recovery within a short time [12,13]. We designed this study to evaluate the 24 hour-effects of early initiation of short term pressure support NIV during the PACU stay in obese day case surgery patients.

## Methods

### Study population

The study was approved by the Ethics Committee of the University of Marburg (AZ 70/08), and informed written consent was obtained. Between 2009/2010 we prospectively included 72 obese adult patients (BMI 30-45, ASA II-III) scheduled for minor peripheral surgery (Table 1). We excluded patients having abdominal surgery or surgery requiring head down tilt. The minimum duration of surgery was set at 45 minutes, maximum of 120 minutes. We also excluded patients with a history of gastro-oesophageal reflux disease, hiatus hernia or requiring emergency operation with rapid sequence induction. Additionally we excluded patients with suspected presence of difficult airway or pre-existing lung impairment (pregnancy, asthma, severe renal dysfunction) as well as cardiac disease resulting in marked limitation of physical activity, corresponding to NYHA class >II, severe psychiatric disorders or difficulties in cooperating during measurements. All were informed about the NIV technique at the pre-anaesthetic visit. They were allocated randomly (adaptive randomization by a study nurse not involved in this study) to a non-invasive ventilation group (NIV-group) (n = 30), or to a control group (n = 30). Patients who did not achieve fast track criteria within 20 minutes after surgery or exhibit adverse events during course were withdrawn from the study.

**Table 1:** Basic data for 60 patients undergoing elective minor peripheral surgery

	*NIV (n = 30)*	*Control (n = 30)*
**Age(yr)**	52 ± 11	53 ± 13
**BMI**	35 ± 3.3	35 ± 2.5
**Surgery time (min.)**	78 ± 26	79 ± 24
**Remifentanil consumption (μg)**	1315 ± 210	1279 ± 199
**Propofol consumption (mg)**	665 ± 122	679.0 ± 143
**BIS-Value during surgery**	49 ± 5.9	46 ± 5.2
**BIS-Value at discontinuation of anesthesia**	62 ± 5.1	63 ± 6.7
**Time to extubation (min.)**	8.3 ± 4.9	9.2 ± 5.3
**fast track score >10 (min.)**	11.2 ± 3.5	10.5 ± 3.9
**Postoperative pritramide(mg) consumption (within 24 h)**	7.5 ± 4.3	8.1 ± 5.8
**Knee Arthroscopy**	n = 12	n = 10
**Minor breast surgery**	n = 10	n = 14
**TUR-Prostate**	n = 5	n = 2
**Hand surgery**	n = 3	n = 4

### General anaesthesia

Twelve hours before surgery patients were premedicated with oral chlorazepat 20 mg. All were pre-oxygenated for 3 minutes at an adjusted fraction of inspired oxygen (FiO_2_) of 1.0. Thereafter anaesthesia was induced with fentanyl 2-3 μg kg^-1 ^and propofol 2 mg kg^-1^

and maintained with remifentanil (0.1-0.2 μg kg^-1 ^ideal body weight) and propofol 3-6 mg kg-1 h-1 [14]. Orotracheal intubation was performed after a single dose of rocuronium (0.5 mg kg^-1 ^ideal body weight); no additional neuromuscular blocking agent was given. A continuous cuff pressure device (Rüsch GmbH, Kernen, Germany) was used to maintain a 30 cmH_2_O cuff pressure by the respective anaesthetist. Standard monitoring was performed throughout (pulse oximetry, non-invasive blood pressure and electrocardiography), plus monitoring of anaesthetic depth levels (BIS EEG, BIS Quatro™; Aspect Medical Systems, Freising Germany). Recovery from neuromuscular blockade was monitored with a peripheral nerve stimulator (TOF-Watch, Organon Teknika Germany) by Train-Of-Four (TOF) ratio (relationship between first and last/fourth neural muscle innervation) assessment to ensure a ratio >0.9 before extubation [15]. During pressure controlled mechanical ventilation, the rate was adjusted to maintain an end-tidal CO2 pressure of 4-4.7 kPa at an inspiration to expiration ratio of 1:1.5 and a positive end expiratory pressure of 10 cmH_2_O. A maximum peak pressure of 30 cmH_2_O was allowed. FiO_2 _during anaesthesia was 0.5. Fifteen minutes before extubation, each patient received dolasetron (25 mg i.v.) and dexamethason (4 mg i.v.) as PONV prophylaxis. The oral cavity was suctioned before extubation. When the patient was fully awake and spontaneously breathing, the trachea was extubated without suction in a head up position with a positive pressure of 10 cmH_2_O at an adjusted FiO_2 _of 1.0. Thereafter patients were transported to the post-anaesthesia care unit (PACU), where they were nursed in the 30°head up position.

### General postoperative Care

Before randomization, blood gas analysis and any signs of postoperative residual curarization (PORC) or opioid overdosage were evaluated by the anaesthetist. A sufficient level of vigilance according to the respective fast-track criteria as well as head up lift for 5 seconds and normal tongue spatula test was achieved in every patient included. The control group received supplemental oxygen via venturi mask at an adjusted oxygen flow of 6 l/min. No additional respiratory treatment or mobilization was performed in either study group during the PACU stay. Blood gas analysis was performed in every patient at each measurement point in the PACU after breathing air for 5 minutes.

### Postoperative pain management

Both groups received basic non-opioid analgesia with intravenous (i.v.) paracetamol 1 g and metamizol 1 g i.v.. Pain was assessed using a visual analogue scale (VAS) at fixed intervals (15 minutes). Analgesia was supplemented with piritramide i.v. whenever the visual analogue scale (VAS) was >4. Overall piritramide consumption was recorded within the first twenty-four hours.

### Non invasive ventilation

Immediately after the first assessment (T0h) pressure support NIV was commenced within the NIV-Group, initially set at 5 cmH_2_O inspiratory pressure, 10 cmH_2_O positive end-expiratory pressure (PEEP) at an adjusted FiO_2 _of 0,5 with each patient in a 30° head-up position using a Dräger "Carina" with the respective NIV full face masks (Dräger AG, Lübeck, Germany). Settings were subsequently titrated according to patient tolerance to achieve an expiratory tidal volume of 6-8 ml^-kg ^ideal body weight. PEEP was tolerated located within a range of 8-10 cmH_2_O; pressure support ranged between 0-10 cmH_2_O. The target duration of the pressure support NIV was 120 minutes.

### Spirometry and pulse oximetry

Spirometry and pulse oximetry were standardized, with each patient in a 30° head-up position [16] after breathing air for 5 minutes. At the pre-anaesthetic visit, a baseline spirometry measurement and pulse oximetry were taken (Tbase) after thorough demonstration of the correct method. Vital capacity (VC), forced vital capacity (FVC), forced expiratory volume in 1 s (FEV1) mid-expiratory flow (MEF25-75), peak expiratory flow (PEF), peak inspiratory flow (PIF) and forced inspiratory vital capacity (FIVC) were measured. At each assessment time, spirometry was performed at least three times to be able to meet the criteria of the European Respiratory Society (ERS) and the best measurement was recorded [17]. In the recovery room (about 5-10 min after extubation), we repeated spirometry (T0) as soon as the patient was alert and fully cooperative (fast track score >10) [18]; pain and dyspnoea were assessed during coughing using the fast track score (>10) before and, if necessary, after analgesic therapy (all patients included in this study met this criterion within 20 min of extubation). Spirometry and pulse oximetry were repeated in the PACU at 1 h (T1) and 2 h (T2). To evaluate the progression of postoperative lung function after PACU discharge, we performed further measurements at 6 h (T3) and 24 h (T4) after extubation. Piritramide requirements were documented prior to each assessment, as soon as the patients were free from pain on coughing. Factors that interfered with breathing (e.g. pain, shivering) were eliminated or at least minimized to produce reliable measurements.

### Statistical analysis and randomization

A prospective power analysis performed with the PASS2002 software (Number Cruncher Statistical Systems, Kaysville, Utah, USA) revealed that 17 patients per group provided a more than 80% chance to detect an absolute improvement of 10 mmHg arterial oxygen partial pressure breathing room air (e.g. 50 mmHg to 60 mmHg) with an expected standard deviation of 10 in both groups using Student's t-test with a type-I error of 5%. To compare postoperative respiratory data and pulse oximetry between the two groups, we tested the null hypothesis (H_0_) that postoperative pulse oximetry values are comparable. In order to demonstrate normal data distribution, a Kolmogorov-Smirnov-Test was performed before each analysis. If normally distributed, a t-test analysis was performed, otherwise the Wilcoxon-Mann-Whitney test was applied. For further characterization we performed repeated-measure analysis of variance (two factor mixed measures analysis of variance) and displayed the interaction between and within the study groups. H_0 _was rejected at an adjusted p of < 0.005 due to multiple testing (Bonferroni). To avoid pre and intraoperative observer bias, randomization was performed after postoperative inclusion criteria were achieved and the first measurements in the PACU were performed. All values of the respective BIS-Index, remifentanil and propofol consumption were collected through an online documentary system (Medlinq Easy Software, Hamburg, Germany). Statistic analysis was by JMP 8 for Windows (SAS Institute Inc., Cary, NC).

## Results

Overall 72 patients were recruited; two declined to continue and measurements were unsatisfactory in a further eight as a result of missed fast track criteria (<10) within 20 min after surgery. Two patients in the control group had laryngo-bronchospasm and were excluded. As a result, we present data for 60 patients (m/w) with 30 individuals per group (Table 1).

The mean duration of surgery was 79 (SD 26) minutes, range 45 -120 minutes. Basic data exhibit no significant differences (Table 1). All patients had been ventilated according to the respective target values and there were no unexpected intubation problems. Antagonism of muscle relaxation was not necessary in any patient. Severe desaturation (SpO_2 _<85%) did not occur in any patient.

### Blood gas analyses

**Figure 1:**
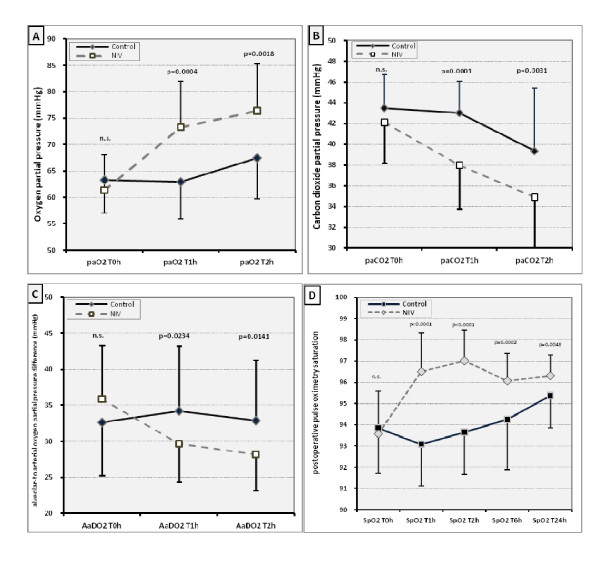
**Postoperative blood gas analyses and pulse oximetry values**. Postoperative pulse oximetry - Difference from preoperative baseline. P-value: t-Test. Interaction between the study groups (ANOVA; p < 0.0001). Bars indicate SD. (n.s.= no significance). All measurements were performed after 5 minutes of breathing room air

In order to control postoperative ventilation and oxygenation we obtained blood gas analysis within fixed intervals in the PACU by radial arterial puncture. At first assessment in the PACU the groups exhibited a similar reduction in oxygen partial pressure. There was no hypercapnia. Overall the NIV-group had significantly better oxygen and carbon dioxide pressures in the PACU (Figure 1 a/b, p < 0.004), and the alveolar to arterial oxygen partial pressure difference (AaDO_2_) was less (Figure 1c, p < 0.03). This improvement was located at T1h for paO_2 _11 ± 8 mmHg (95% CI 6 to 14 mmHg), paCO_2 _5 ± 3 mmHg (96% CI 3-6 mmHg), AaDO_2 _5 ± 9 mmHg (95% CI 2-9 mmHg), at T2h for paO_2 _9 ± 7 mmHg (95% CI 6-12 mmHg), paCO_2 _4 ± 3 (95% CI 3-6), AaDO_2 _5 ± 8 mmHg (95% CI 2-9 mmHg).

### Pulse Oximetry

Baseline (preoperative) pulse oximetry values were within the normal range of 95-99%; there were no differences between groups before or after premedication (Table 2). In both, the lowest values were found directly after extubation, in the PACU, after achieving a fast track criteria value >10. At the first measurement point in the PACU before initiation of pressure support NIV, there was no difference between groups. Overall pulse oximetry saturation ranged between 99 and 85% (mean 94%) at first assessment in the PACU. During the first hour in PACU (T0h-T1h; p < 0.0001, Figure 1d) the NIV group had better pulse oximetry values than the controls (ANOVA p < 0.0001), and also thereafter at 6 h and 24 hours after surgery (Figure 1d; p < 0.005). Overall mean pulse oximetry values differed by three percentage points.

**Table 2:** Preoperative lung function and pulse oximetry saturation baseline values breathing room air (n.s. = no significance)

	*SpO2(%) before premed.*	*SpO2(%) after premed.*	*FVC (l)*	*FEV1 (l)*	***PEF ***(l/s)	***MEF 25-75 ( ***l/s)
**NIV**	96.9 ± 1.0	95.8 ± 1.5	3.81 ± 1.1	3.05 ± 0.9	6.9 ± 1.9	3.15 ± 1.2
**Control**	97.0 ± 1.4	95.9 ± 1.9	3.69 ± 1.0	2.98 ± 0.7	6.8 ± 2.3	3.09 ± 1.1

**t-test p < 0.05**	n.s.	n.s.	n.s.	n.s.	n.s.	n.s.

### Spirometry

**Figure 2:**
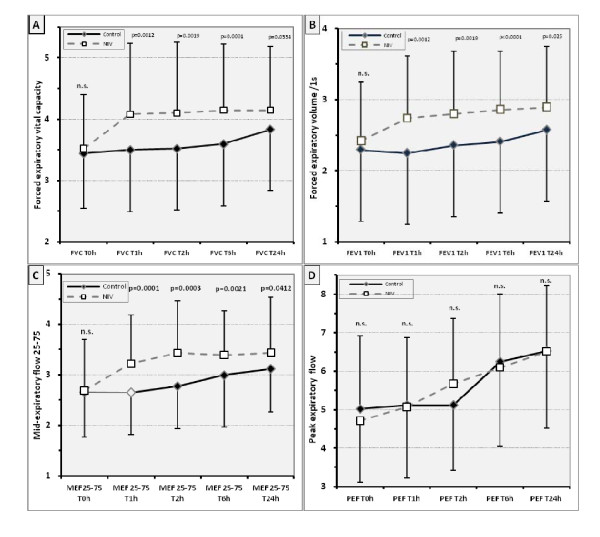
**Postoperative lung function measurements (FVC/FEV1/MEF25-75/PEF) - Bars indicate SD. n.s.=no significance**. For abbreviations, see text.

Preoperative in- and expiratory spirometry values (baseline) were within the normal range between the upper limit of normal and the lower limit of normal (LLN) as previously described (Table 2) [19]. Postoperative spirometry values, except of peak inspiratory, showed a similar pattern to pulse oximetry (Figure 2). The NIV group recovered lung function faster during the PACU stay, almost reaching preoperative baseline values (Figure 2; p < 0.001-between interaction) while the control group recovered in- and expiratory lung volumes only moderately. This time effect (T0h-T1h; p < 0.0001) within the study groups ceased during course. Even after the first postoperative mobilisation and on the first day after surgery, lung function values in the controls were up to 20% below baseline (Figure 2 p < 0.05). Although the overall difference in lung function between groups had decreased 24 hours after surgery, significant differences still remained.

### Postoperative management

No patient experienced severe postoperative pain; the maximum VAS scale pain score before analgesia was six in both groups. Opioid consumption for the first 24 hours was comparable in the two groups (Table 1). At each measurement, every patient had an acceptable level of vigilance, and no pain. Shivering or nausea, which might have interfered with spirometry, was not present in any patient.

## Discussion

Atelectasis and respiratory impairment are common after general anaesthesia [20,21]. Perioperative atelectasis occurs within minutes, mostly due to compression and oxygen reabsorption caused by a high fraction of inspired oxygen (FiO_2_) [22]. A high FiO_2 _is commonly used at the discontinuation of general anaesthesia, although its detrimental effects on lung function are well known [23,24]. Hypoventilation as well as lack of vigilance may also contribute to early postoperative lung impairment.

Several studies have documented the favorable effects of NIV in acute respiratory failure, mostly in an intensive care setting. This suggests that many trials of NIV are required, at times uninterrupted for several hours, to achieve positive results [25-29]. Our data show that even short term NIV in the PACU when commenced immediately after extubation is sufficient to enhance pulmonary function for the following 24 hours at least.

Overall differences were small and pulse oximetry measurement as well as lung function measurement has a certain bias [30,31]. Such bias would affect both study groups, but cannot be completely ignored. The measured effect may be altered due a lack of preselecting in terms of risk factors e.g. obstructive sleep apnoea (OSA). This may reinforce the overall magnitude of the effect of a short term NIV.

There is no question that NIV and CPAP improve gas exchange, minimize atelectasis, and increase functional residual capacity [4,32,33]. Previous findings indicated that early, pre-emptive, initiation of either mode reduces overall pulmonary complications, although study objectives and overall treatment times varied greatly [25,26,34]. In contrast, short term approaches or late initiation are of no benefit [35,36]. An alternative approach using chest physiotherapy proved beneficial in terms of pulmonary function, although not clearly superior to NIV [37], and staff costs may have been larger than using technical devices.

What else may be responsible for our findings? Postoperative vigilance may be important, and the painful stimulus of blood gas collection may have altered postoperative wakefulness; fast track scores are too crude for a detailed evaluation of postoperative consciousness. Lingering drug effects may promote increased upper airway collapse in the obese [38]. Postoperative residual curarization (PORC) [39,40], which cannot be excluded even when neuromuscular monitoring is used, may have a greater impact in the obese. This hypothesis is supported by our expiratory peak flow measurements which exhibit no significant differences between groups while displaying similar (temporary) limitation of inspiratory flow within the first postoperative hour (additional files. Drug effects are more marked after short procedures and are aggravated with increasing age [40,41]. Especially upper airway muscle tone can be negatively affected by propofol [42].

We found short term NIV beneficial, possibly because it was commenced earlier than in studies performed following different types of surgery and in a population of normal weight. NIV may have been more beneficial in our obese patients as the associated respiratory changes are predominantly based on loss of functual residual capacity. NIV ventilation with PEEP attenuates these effects if initiated early after extubation, increases alveolar ventilation and improves gas exchange. The effect was not due to supplemental oxygen, as measurements were performed five minutes after discontinuation of NIV/supplemental oxygen.

Even mobilisation does not completely abolish post-operative respiratory changes [43]. Thus it seems feasible that short term NIV is beneficial and has a lasting effect, most probably due to increasing functual residual capacity, improved alveolar ventilation and avoidance of atelectasis. Anyhow overall improvements are small and clinical significance has to be established. In this regard patient selection has to be improved. It is not clear whether therapeutic benefit would be obtained after major surgery or in patients with OSA [44]. Nevertheless, short term NIV seems to be a reasonable therapeutic option in the obese after general surgery. As day case surgery becomes more frequent even for the morbidly obese, it may become more necessary.

## Conclusion

Early initiation of short term NIV during in the PACU promotes more rapid recovery of postoperative lung function and oxygenation in the obese. The effect lasted 24 hours after discontinuation of NIV. Patient selection is necessary in order to establish clinically relevant improvements.

## Limitations

As indicated within the methods section, overall pulmonary function testing was not blinded and thus a bias may be possible. As preoperative blood gas analysis was not allowed by the ethics committee, preoperative differences between the study populations cannot be excluded. Another major limitation factor is the pre-selection of our obese patients scheduled for minor peripheral surgery. Only 11 of these (6 NIV-group, 5 Control-group) were morbidly obese. Moreover, no operations with abdominal insufflations (laparoscopy) or head down tilt were included, nor any patients with gastro-oesophageal reflux disease or hiatus hernia, which may limit the applicability of our results for major surgery. Nevertheless major surgery, especially abdominal, affects respiratory mechanics to a greater degree and there is more post-operative pain. The primary aim of our study was to examine the impact of NIV on lung volumes in the obese in the immediate postoperative period, when the impacts of surgical trauma and anesthesia are most likely to trigger pulmonary morbidity. Therefore using this study design we minimized possible factors interfering with our measurements.

NIV requires trained nurses and initial equipment expenses, and criteria for its prophylactic use in the immediate postoperative period have not yet been defined. Additionally, we do not know whether NIV is superior to CPAP alone. Furthermore we cannot draw any conclusion about clinical outcome or other gold standards such as the incidence of pneumonia. Large scaled studies, especially on day cases, and focussing on patients at risk for postoperative pulmonary complications (e.g. OSA) are required.

## Competing interests

The authors declare that they have no competing interests.

## Authors' contributions

MZ (MD,DEAA) - conceived of the study and participated in its design and coordination, drafted the manuscript, participated in performing the statistic analysis, read and approved the final manuscript. GK (MD) - carried out the measurements, participated in coordination of the study and drafted the manuscript, read and approved the final manuscript. DB (cand. med.) - carried out the measurements, participated in coordination of the study, read and approved the final manuscript. LE (MD) - participated in its design and performed the statistic analysis, read and approved the final manuscript. NZ (MD,PhD) - participated in its design and drafted the manuscript, read and approved the final manuscript. HW (MD) - drafted the manuscript, read and approved the final manuscript. FD (MD) - participated in its design and coordination, carried out measurements, drafted the manuscript, read and approved the final manuscript

## Pre-publication history

The pre-publication history for this paper can be accessed here:

http://www.biomedcentral.com/1471-2253/11/10/prepub
